# Geodemographic Patterns of Meat Expenditure in Great Britain

**DOI:** 10.1007/s12061-020-09344-5

**Published:** 2020-06-16

**Authors:** William H.M. James, Nik Lomax, Mark Birkin, Lisa M. Collins

**Affiliations:** 1 School of Geography and Leeds Institute for Data Analytics, University of Leeds, Woodhouse Lane, Leeds, West Yorkshire LS2 9JT UK; 2 Faculty of Biological Sciences, University of Leeds, Woodhouse Lane, Leeds, West Yorkshire LS2 9JT UK

**Keywords:** Geodemographic classification, Expenditure patterns, Diet, Health, Environment

## Abstract

The future of the meat industry will require the management of important trade-offs between economic, environmental and health aspects of both humans and animals. Understanding the patterns and trends of meat expenditure and consumption is crucial for assessing the current resilience of the system and for economic, planning, health and environmental applications. Here, we show how the technique of geodemographic classification, combined with fine scale expenditure estimates can be used to explore temporal and spatial patterns of meat expenditure in Great Britain between 2008 and 2017. Whilst the expenditure patterns of some food categories such as sausages remained relatively consistent, others such as lamb show a trend towards a reduced proportion of expenditure and increased inequality of purchases. Short term changes in expenditure patterns also occurred, potentially due to product specific price variability, price elasticities or zoonotic disease scare. Environmental attitudes, financial constraints and the prominence of communities who do not eat meat for religious or cultural reasons are likely to be driving the differences between geodemographic groups. The methodology and results could be a valuable tool for policy makers in the meat industry and beyond.

## Background & Summary

The meat industry is an important and often contentious component of the food system, with ongoing debate surrounding animal welfare, environmental impacts, human health and economic importance. The UK meat processing industry directly employs over 75,000 people and supports more than 50,000 farmers (BMPA 2018), producing over £8 billion of meat in 2018 (DEFRA 2019). Although meat is a source of nutrients that are important components of a balanced diet (Wyness 2016), there is a body of evidence that links the over-consumption of processed and red meat to an increased risk of obesity and diseases such as cancer, diabetes and heart disease (Micha et al. 2012; Rouhani et al. 2014; WCRF 2017). The environmental impact of the industry is also commonly raised as a concern, with meat production generally producing more emissions per unit of energy compared with plant-based foods because energy is lost at each trophic level (Gerber et al. 2013). However, this is a complex issue as meat production may or may not compete for resources that could be used to produce alternative food types, and because it depends critically on how harm to the environment is measured (Godfray et al. 2018). Environmental impacts also vary by livestock type, with production of 1 kg of beef generally having the highest global warming potential and requiring the most land and energy. This is followed by production of 1 kg pork and chicken (De Vries and de Boer 2010). Within this, there is considerable variability due to location specific factors. For example, favourable conditions for grass fed systems result in UK-produced beef having approximately half the emissions of the global average (Committe on Climate Change 2020). These environmental concerns are recognised by the UK meat industry, with the National Farmers Union aiming to achieve net zero greenhouse gas emissions across the whole of the agriculture sector in England and Wales by 2040 (NFU 2019). Whilst this presents significant challenges for the UK meat industry, there are a variety of technologies and practices available to minimise the trade-offs between environmental and economic concerns (Gerber et al. 2013). For example, methane emissions from livestock can be reduced by using feed additives and generally improving animal health, whilst gene editing offers the possibility of improved productivity and reduced emissions (NFU 2019). To tackle the trade-offs between health, environmental and economic factors, it is crucial to understand the patterns and trends of meat expenditure and consumption. This will offer insights into the resilience of the system and for economic, planning, health and climate change applications.

Over the past 50 years, the UK has experienced major shifts in dietary patterns due to changes in agricultural practice, trade policies and food industry marketing (Kearney 2010). Most recently, veganism, vegetarianism and flexitarianism has attracted attention in the literature and popular media, with a number of reports (e.g. Willett et al. 2019) and celebrities (Phua et al. 2019) advocating conversion to diets with reduced meat content. Further changes may also be on the horizon in the context of a UK exit from the European Union (DEFRA 2018). Against this backdrop of continuous national level change, there is substantial local level variability in meat consumption and expenditure due to the spatial variation of demographic and socioeconomics drivers. Religion is a good example of this, where an individual’s beliefs may restrict or forbid meat consumption (Shatenstein and Ghadirian 1998). Other drivers include culture (Haverstock and Forgays 2012), gender and socioeconomic status (Clonan et al. 2015). By understanding the spatial and sub-group variation of these drivers, it is possible to improve our knowledge of the resilience of the industry and to help inform future projections of both demand and production. This will aid policy makers in targeting the correct sociodemographic groups as the industry strives to achieve sustainability and profitability whilst meeting the evolving and complex demands of consumers and organisations. Targeted policy interventions aimed at certain sociodemographic groups are already used under a range of circumstances, often to achieve sustainability or health outcomes. For example, Minimum Unit Alcohol Pricing in Scotland aims to benefit health outcomes in harmful drinkers (Angus et al. 2016), whilst UK Vehicle Excise Duty is designed for environmental outcomes by heavily taxing owners of highly polluting luxury cars (DVLA 2019).

The primary aim of this study is to investigate the current and recent resilience of the UK meat industry by examining temporal and spatial patterns of expenditure. In the context of this study, aspects of resilience include the ability to develop resistance and foster recovery in response to extreme events (Béné et al. 2014) and refers to coping mechanisms and adaptive capacities that provide the means to overcome exposures and sensitivities associated with vulnerabilities (Doherty et al. 2019). Utilising newly available datasets of meat expenditure, this study shows how the technique of geodemographic classification can be used to help assess the resilience of the meat industry by dissecting consumer behaviour based on where they live and their sociodemographic characteristics. Moving away from a homogeneous view of the consumer is important for understanding how trends and preferences vary for these different groups. We also utilise YouGov survey data of consumer attitudes to investigate the links between patterns of meat expenditure and perspectives on environmental issues and diet.

This research is motivated by the PigSustain research project, aiming to assess the resilience of the UK pig industry historically, currently and into the future. Due to the interconnected nature of the meat industry, this study takes a holistic approach by investigating a range of meat products and meat alternatives.

## Methods

### Expenditure Data

This research utilises expenditure estimates for various meat and associated categories at the Local Authority District Level for Great Britain (for 380 geographical areas) generated by James et al. (2019). These data were constructed using Spatial Microsimulation, linking individual expenditure survey data from the Living Cost and Food Survey (LCFS) with a variety of other local level data from the 2011 Census of population and other sources. The original data covers the time period 2008–2016 and this study updates the repository to include results for 2017 (the latest available iteration of the LCFS). The full methodology for generating the data can be found in James et al. (2019). The data used accounts for expenditure by residential households, not institutions (e.g. hospitals and prisons), however analysis of the National Diet and Nutrition Survey (NDNS 2019) found that approximately 80% of meat consumption is accounted for by purchases for household supply.

This study focusses on nine food categories which are of relevance to the meat sector. The meat categories are pork, bacon and ham, sausages, beef, lamb and poultry while non-meat products are analysed under the categories fruit and vegetables and other processed products. Appendix 1 Table 2 provides further detail of the range of specific products covered by each. Analysis includes both meat and non-meat categories because consumers are known to substitute one product for another due to factors such as a price rise or disease scare in a particular meat (e.g. Pritchett et al. 2007). The category ‘other preserved or processed vegetables’ contains, amongst others, typical meat substitutes such as soya & novel protein foods which are typically associated with providing a non-meat protein source, especially in vegetarian and vegan diets.

### Geodemographic Classification

Knowledge of an area’s demographic characteristics is important for understanding the needs of those who live in, work or service the area (Clark et al. 2017). In the context of the meat industry, it is important for assessing the market resilience of the area and planning for related infrastructure and resources (e.g. processing facilities, commercial premises, healthcare provision). Whilst every area is unique, some will be very similar to others and some will be distinctly different. Geodemographic classification is the spatially explicit classification of socio-economic data and has been successfully used in the fields of health (Abbas et al. 2009), crime (Ashby and Longley 2005) and education (Singleton and Longley 2009). It can be used as a powerful commercial tool in the fields of market research, market analysis, direct marketing and advertising (Harris et al. 2005).

Whilst bespoke classification solutions can be developed using selected input data for specific research needs (e.g. Clark et al. 2017), there are a number of ‘off the shelf’ products which can be used to identify areas with similar sociodemographic characteristics. Within the UK, the Office for National Statistics (ONS) publishes a freely available suite of classifications based on census data (Gale et al. 2016). Although the fundamental input data varies between geodemographic classifications, they generally follow the same basic workflow; data acquisition, data manipulation and transformation and cluster analysis (typically of a k-means variant). For further details of clustering and in particular the k-means approach the reader is directed to Burns (2017) and Vickers and Rees (2006).

**Fig. 1:**
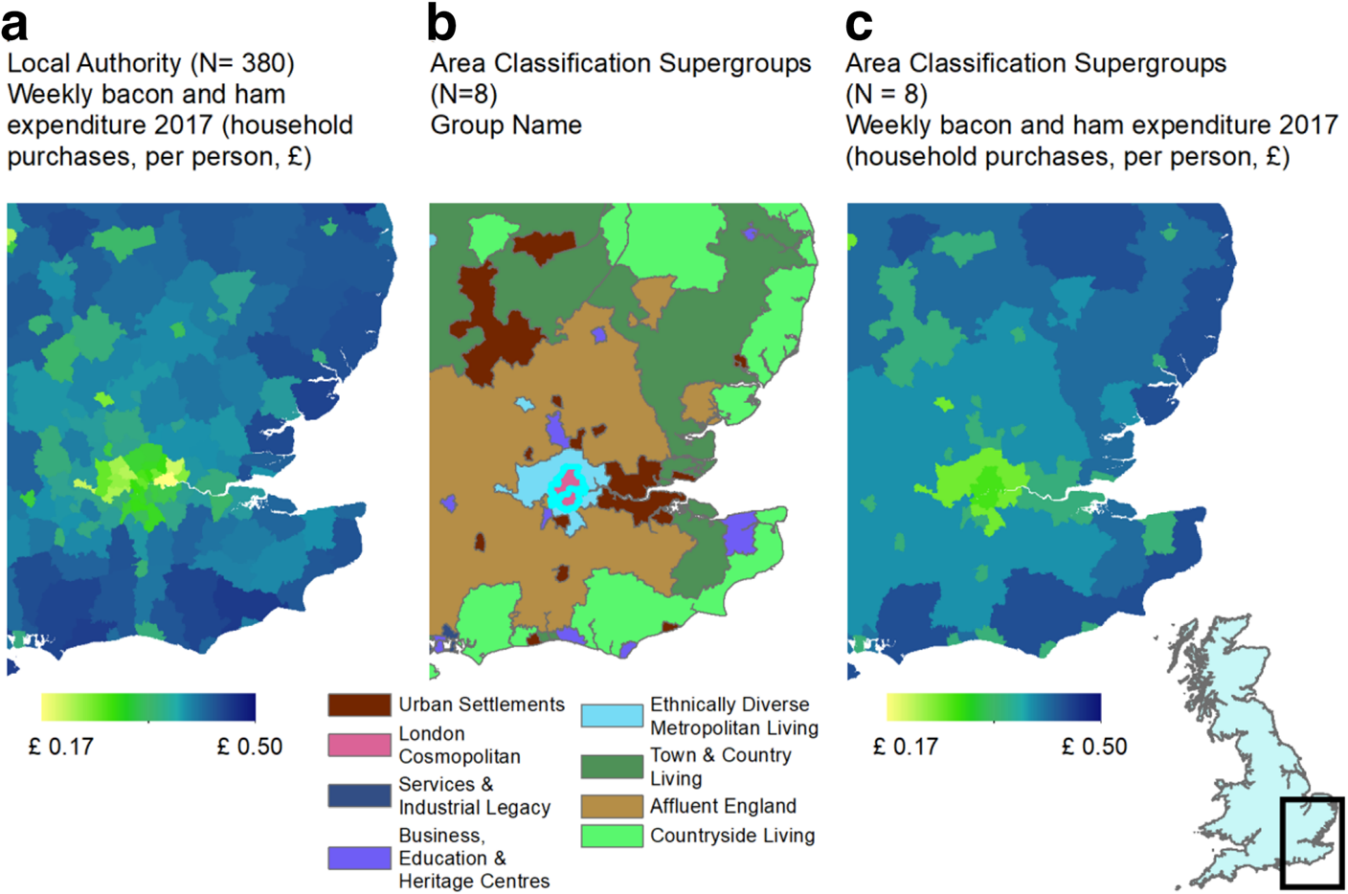
**a** Initial expenditure data (£ per person/week) from James et al. (2019). **b** Super-group classification for Great Britain. **c** Derived expenditure estimates at the super-group level

With the expenditure data of this study at the Local Authority District Level (Fig. 1), we use the ONS area classifications based on the same geography (Fig. 1b). This allows us to generate an estimate of expenditure for each geodemographic group, as exemplified in Fig. 1c. As the expenditure estimates used in this study cover the years 2008 to 2017, we use the 2011 classifications (the latest available) which were downloaded from https://www.ons.gov.uk/methodology/

The 2011 Area Classification for Local Authorities (Fig. 1b) is based on a suite of 59 census statistics and a K-means clustering technique. The statistics are sourced from the domains of demographic structure, household composition, housing, socio-economic and employment as listed in Appendix 2 Table 3. The classification scheme has a three-tiered hierarchical structure, separating Local Authority Districts into super-groups (*n* = 8), groups (*n* = 16) and sub-groups (*n* = 24), as shown in Table 1. This gives us the flexibility to investigate expenditure patterns at a range of scales. Each group has an associated name and ‘pen portrait’ to represent the underlying complexity of the cluster composition, as described in Appendix 3.

**Table 1:** Name and hierarchical structure of the classification scheme

Super-groups (*n* = 8)	Groups (*n* = 16)	Sub-groups (*n* = 24)
1r: Affluent England	1ar: Rural-urban fringe	1a1r: Rural-urban fringe
	1br: Thriving rural	1b1r: Affluent rural
		1b2r: Rural growth areas
2r:Business, education and heritage centres	2ar: Larger towns and cities	2a1r: Larger towns and cities
	2br: University towns and cities	2b1r: University towns and cities
3r: Countryside living	3ar: English and Welsh countryside	3a1r: Older farming communities
		3a2r: Sparse English and Welsh countryside
	3br: Remoter coastal living	3b1r: Ageing coastal living
		3b2r: Seaside living
	3cr: Scottish countryside	3c1r: Scottish countryside
4r: Ethnically diverse metropolitan living	4ar: Ethnically diverse metropolitan living	4a1r: Ethnically diverse metropolitan living
5r: London cosmopolitan	5ar: London cosmopolitan	5a1r: London cosmopolitan
6r: Services and industrial Legacy	6ar: Services, manufacturing and mining legacy	6a1r: Manufacturing legacy
		6a2r: Mining legacy
		6a3r: Service economy
	6br: Scottish industrial legacy	6b1r: Scottish industrial legacy
7r: Town and country living	7ar: Country living	7a1r: Country living
	7br: Northern Ireland countryside	7b1r: Northern Ireland countryside
	7cr: Town living	8a1r: Prosperous semi-rural
		8a2r: Urban living
8r: Urban settlements	8ar: Manufacturing traits	8b1r: City periphery
	8br: Suburban traits	8b2r: Expanded areas

### Utilising Geodemographic Segmentation to Assess the Resilience of the Meat Industry

As raw expenditure values will vary depending on inflation, we standardise the data by calculating expenditure as a proportion of total household food expenditure. We also calculate the equality of expenditure for each product category and for each year. This is a useful measure as it can give an insight of the adaptive capacity and means to overcome vulnerabilities, both key aspects of food system resilience (Doherty et al. 2019). If a market share is dominated by a small proportion of individuals (whilst the remaining population do not contribute any expenditure), the system may have a low resilience as there is no adaptive capacity if an event such as a health scare or societal change reduces the expenditure of the few individuals. Conversely, if there is equality of expenditure (i.e. all members of the population contribute an equal expenditure), the system may be better able to adapt and re-organise if expenditure is reduced by any segment of the population.

**Fig. 2:**
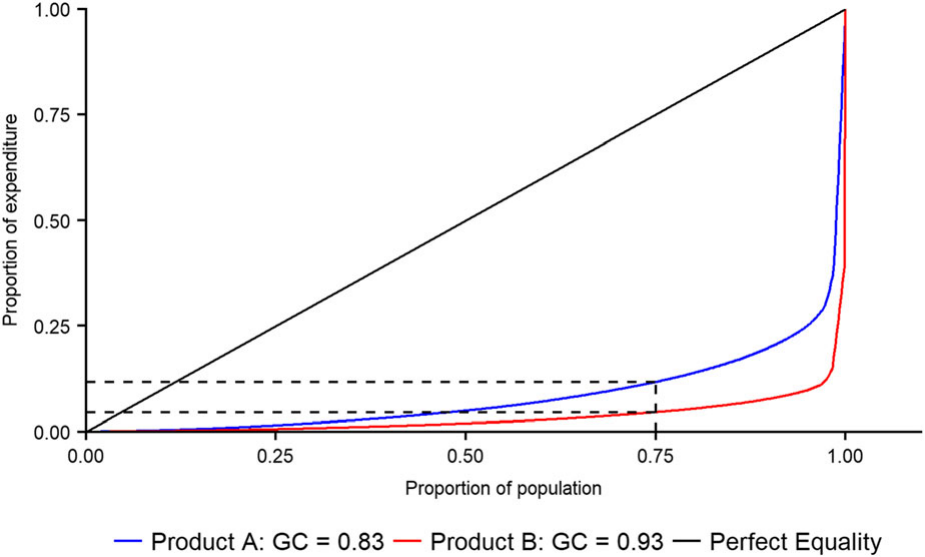
Lorenz curves and Gini coefficients for two hypothetical products

To quantify the equality of expenditure, we use the Gini Coefficient (Gini 1912), hereafter termed GC. This statistic is traditionally used by economists and sociologists to measure economic inequality (e.g. Roemer 2013) by condensing the distribution of a variable into a single value between 0 and 1. A GC of zero expresses perfect equality (everyone has the same expenditure) whilst a GC of 1 denotes maximal inequality (a single person is responsible for all expenditure whilst everyone else has zero expenditure). The GC is derived from a Lorenz Curve, as demonstrated in Fig. 2. The GC is equal to the area between the distribution curve (the red or blue line in Fig. 2) and the line of perfect equality (the black line in Fig. 2), scaled between 0 and 1. Figure 2 shows how hypothetical product A (blue line) has relative equality of expenditure (GC = 0.83) compared to product B (red line, GC = 0.93). The curve can also be used to estimate the proportion of expenditure which any given proportion of the population is responsible for. The dashed lines on Fig. 2 show that 75% of the population is responsible for 12% of the expenditure for product A, whilst accounting for just 5% of the expenditure of product B.

By using the geodemographic classifications discussed above, we are able to calculate individual GC’s for each geodemographic group in a similar manner to the ‘area-based Gini coefficient’ technique of Druckman and Jackson (2008). The time series nature of the expenditure data also allows us to measure temporal trends in GC for each group, allowing us to assess trends in expenditure inequality.

### Consumer Attitudes and Expenditure Inequality: YouGov Survey Data

With a recent increase in awareness of the role of the livestock industry in relation to climate change (e.g. Rijsberman 2017) and of the potential health risks of excessive meat consumption (e.g. Rouhani et al. 2014), we utilise YouGov survey data to investigate the links between expenditure patterns and consumer attitudes. The YouGov survey was carried out in 2015–16 and provides data on consumer attitudes for a sample of 250,000 adults across the UK. We focus on four questions related to climate change and health (and thus of relevance to the meat industry). Responses are given on a five-point scale, from ‘definitely agree’ to ‘definitely disagree’. The survey asks for a response to the following statements: ‘Climate change is the biggest threat to civilization’, ‘I consider myself to be a healthy eater’, ‘I don’t mind paying more for products which are good for the environment’ and ‘I don’t care what my carbon footprint is’. These data are reported at the Local Authority scale, so can be aggregated to geodemographic groups in the same manner as the expenditure data.

## Results

### Geodemographic Segmentation of Expenditure Data

**Fig. 3:**
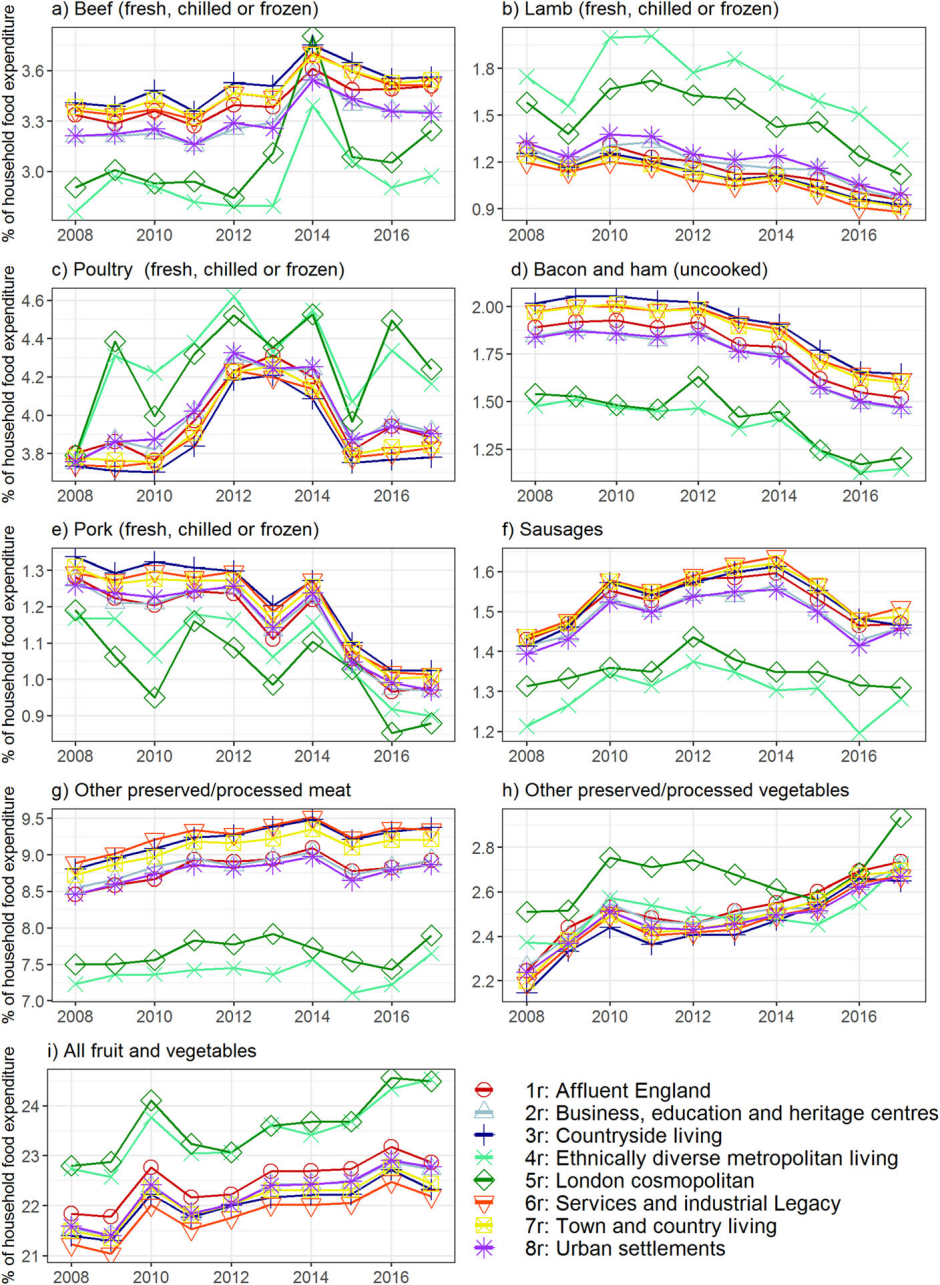
Percentage of total food expenditure separated by product category and geodemographic groups for 2008–2017

**Fig. 4:**
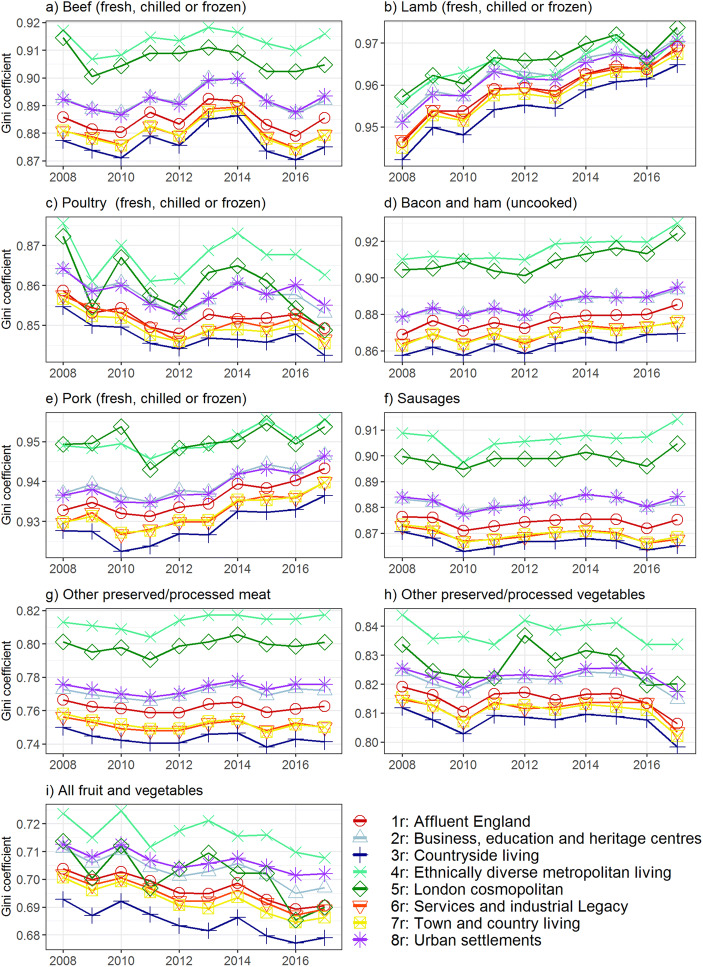
Gini coefficients separated by product category and geodemographic groups for 2008–2017

**Fig. 5:**
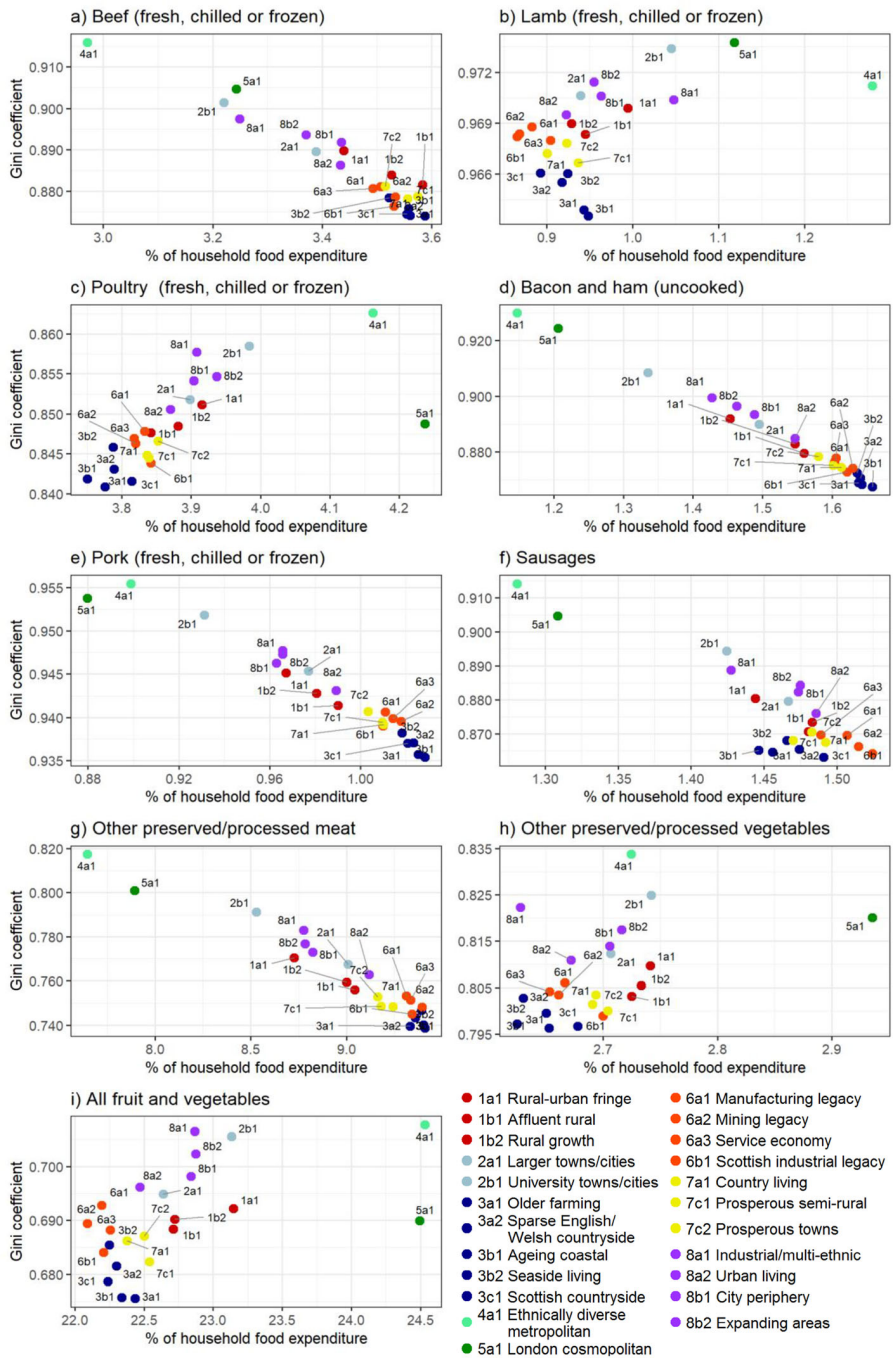
Gini coefficient and % of household food expenditure for each product category (2017)

For each year and for each of the nine product categories, the percentage of total household food expenditure (Fig. 3) and GC (Fig. 4) was calculated. To provide a meaningful overview, results presented here are at the super-group level (*n* = 8), although they could be generated for the sub-group or group level if required. For the year 2017 (the most recent year for which data is available), GCs were plotted against the percentage of total household food expenditure for each product category, with results displayed to the sub-group level in Fig. 5.

Figures 3 and 4 show there is clear variation between product categories in both percentage of expenditure and GC. For example, in 2017 lamb accounted for approximately 1.2% of total household food expenditure (Fig. 3b) and had a GC of ~0.97 (Fig. 4b). Conversely poultry accounted for over 3.7% of the household food budget (Fig. 3c), with expenditure more evenly distributed (GC ~0.86; Fig. 4c). Some product groups exhibit a clear temporal trend of expenditure and GC, whilst others remain relatively constant throughout the study period. A notable example is lamb, which shows a negative trend in percentage of expenditure (Fig. 3b) and positive trend in GC (Fig. 4b).

Geodemographic segmentation reveals differences in expenditure patterns between groups. Super-groups 4r (Ethnically diverse and metropolitan living) and 5r (London cosmopolitan) exhibit relatively low expenditure and low GCs for the red and processed meat categories of beef (Fig. 3/4a), bacon and ham (Fig. 3/4d), pork (Fig. 3/4e), sausages (Fig. 3/4f) and other preserved/processed meats (Fig. 3/4 g). In contrast, geodemographic groups such as 3r (Countryside living) display generally higher percentages of expenditure and lower GC values for these product categories. For example, in 2017 super-group 4r spent on average 1.28% of their food budget on sausages with relative expenditure inequality (GC = 0.915) whilst super-group 3r spent 1.45% with more equal expenditure (GC = 0.865). Whilst the GCs of super-groups 4r and 5r are high across all categories (Fig. 4), these geodemographic groups spend a greater proportion of their food budget on lamb (Fig. 3b), poultry (Fig. 3c) and non-meat categories (Fig. 3h & i) compared to other geodemographic groups.

Superimposed upon the general trends are short term fluctuations in expenditure and GC. For example, the proportion of expenditure for poultry increases between 2010 and 2013 before returning to pre-2010 levels (Fig. 3c). Fluctuations are also visible in GC, with beef (Fig. 4a) exhibiting a peak in 2013–14 before returning to previous levels. Fluctuations are often more evident in certain geodemographic groups than others, with the 2013–14 GC beef fluctuation clearly visible in groups such as 3r (Countryside living) whilst much less prominent for group 4r (Ethnically diverse and metropolitan living).

Figure 5 shows the correlation between GC and the proportion of total food expenditure in 2017. Many red and processed meat categories exhibit a negative correlation (i.e. geodemographic groups which spend a greater proportion of their food budget on these products also tend to exhibit more equal expenditure). These categories include beef (r = −0.96, Fig. 5a), bacon and ham (r = −0.99, Fig. 5d), pork (r = − 0.97, Fig. 5e), sausages (r = −0.87, Fig. 5f) and other preserved/processed meats (−0.96, Fig. 5g). Conversely, other products display a positive correlation, such as lamb (r = 0.54, Fig. 5b), poultry (r = 0.67, Fig. 5c), other preserved/processed vegetables (r = 0.43, Fig. 5h) and all fruit and vegetables (r = 0.53, Fig. 5i). There are outliers, for example group 5a (London cosmopolitan) exhibits the highest proportion of expenditure on chicken for any geodemographic sub-group (4.24%, Fig. 5c), whilst having a relatively low GC (0.848).

### Geodemographic Segmentation of YouGov Survey Data

**Fig. 6:**
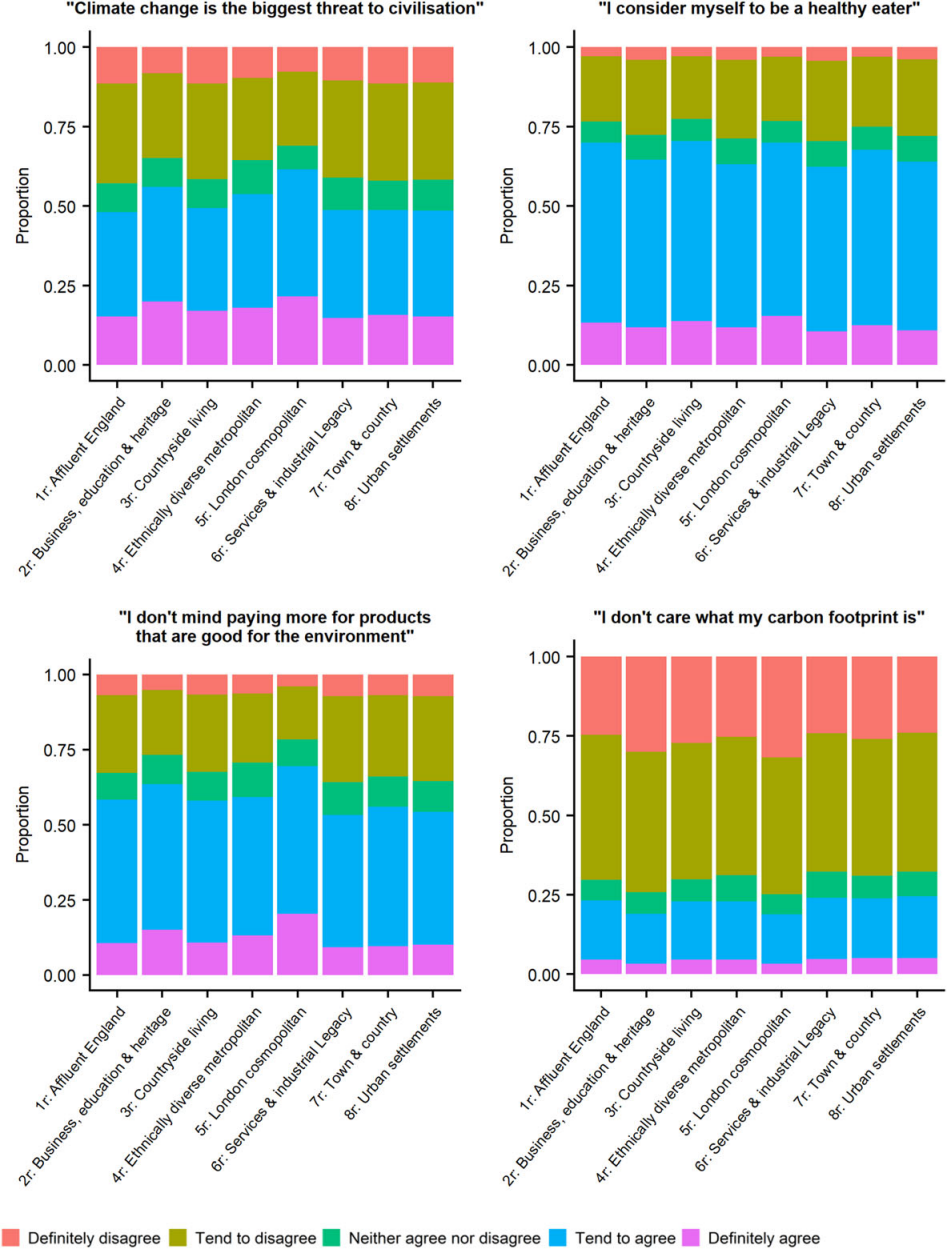
Responses of YouGov survey questions used in this study, aggregated by geodemographic super-group

Figure 6 shows how the results from the YouGov survey can be aggregated to geodemographic groups, allowing comparison of expenditure patterns and consumer attitudes. There is variation between geodemographic groups, with group 5r (London cosmopolitan) demonstrating the most environmentally friendly responses. This group has the highest proportion of respondents answering favourably to the questions ‘Climate change is the biggest threat to civilisation’, ‘I don’t mind paying more for products which are good for the environment’ and ‘I don’t care what my carbon footprint is’. This group also has the second highest proportion (70%) of respondents believing they have a healthy diet (answering ‘definitely agree’ or ‘tend to agree’ to the question ‘I consider myself to be a healthy eater’).

## Discussion

The results presented in Fig. 3–5 demonstrate how geodemographic classification can successfully be used to investigate expenditure patterns for meat products and alternatives. Care should be taken when interpreting these expenditure data as a change in proportion of expenditure does not always correlate with a change in actual consumption (e.g. grams/kg consumed). Product level inflation rates and price elasticities may alter the expenditure patterns (Tiffin et al. 2011), with consumers potentially switching to similar products at different price-points. There is also a range of consumption behaviours within each demographic group that cannot be captured with the data which are available. For example, expenditure patterns may differ between outlet types, such as between supermarkets and farmers markets (Jilcott et al. 2011). Nevertheless, the metrics of proportion of expenditure and GC can be used to assess the resilience of the industry and to compare the expenditure patterns between different geodemographic groups.

Striking differences between expenditure patterns can be seen in red and processed meat categories (specifically beef; Fig. 3/4a, bacon; Fig. 3/4d, pork; Fig. 3/4e, sausages; Fig. 3/4f and other processed meats; Fig. 3/4 g) where super-groups 4r (Ethnically diverse and metropolitan living) and 5r (London cosmopolitan) spend relatively little of their food budget whilst exhibiting relative inequality of expenditure compared to other geodemographic groups. Results from the YouGov survey (Fig. 6) suggests attitudes towards the environment and health may partly explain these differences, especially for group 5r which has the most environmentally friendly and health aware attitudes of any geodemographic group. This explanation is less likely for group 4r (Ethnically diverse and metropolitan living) which displays less environmentally friendly attitudes and lower health awareness. In this case, cultural and religious beliefs may be a more important driver of meat expenditure patterns as a major characteristic of this group is a high proportion of residents from non-White ethnic groups (Appendix 3). This may result in a lower proportion of expenditure and a higher GC as certain ethnic and religious groups consume little or no meat. Specifically, devout Hindus and Buddhists eat no meat at all whilst Islam and Judaism forbid the consumption of pork products (Shatenstein and Ghadirian 1998). Unemployment in group 4r is also higher than the national rate (Appendix 3), potentially also explaining the low proportion of expenditure and high GCs as some residents are unable to afford meat products due to financial constraints. These factors show how multiple drivers need to be considered, and how similar expenditure patterns may be due to a variety of different factors.

The time series nature of Figs. 3 and 4 offers an insight into how meat expenditure patterns have changed between 2008 and 2017. An example of this is lamb (Fig. 3e/Fig. 4e), exhibiting a trend towards reduced proportion of food expenditure and increasing inequality of expenditure. This may be due to shifting dietary preferences, with the National Farmers Union and National Sheep Association stating an ‘aging consumer market’ as one of the weaknesses of the British lamb industry (NSA 2014). This is evident when considering the age structure of the geodemographic groups, with expenditure falling most rapidly in younger groups. For example, between 2008 and 2017 the proportion of expenditure on lamb for the youngest geodemographic group 5r (London Cosmopolitan, median age 32 years) fell by 0.46%, whilst for group 3r (Countryside Living, median age 46) it fell by just 0.33%. Superimposed upon the general temporal trends are short term fluctuations of proportion of expenditure and GC. A notable example is poultry where the proportion of expenditure increases between 2010 and 2013 before returning to pre-2010 levels (Fig. 4c). Fluctuations are also visible in the GC dataset, with beef (Fig. 5a) exhibiting a peak in 2013–14 before returning to previous levels. These fluctuations could be due to the complex interaction of product level inflation rates and price elasticities, leading to consumers substituting one product for another. Disease and health scares within the meat industry may also cause fluctuations in the expenditure patterns as some consumers temporarily restrict their spending on certain products (Pritchett et al. 2007), with the horse meat scandal of 2013 (O’mahony 2013) a potential cause of the 2013–14 GC fluctuation observed in beef (Fig. 3a).

Although the YouGov data used in this study is cross-sectional in nature and therefore does not provide any information on changes in attitudes over time, there is evidence from other sources to suggest an increasing awareness of health and environmental issues related to the meat industry (e.g. Frank et al. 2017). This is also evident in traditional and social media, with a number of high profile celebrities recently endorsing veganism and plant based diets (Phua et al. 2019). These changing attitudes may be expected to influence meat expenditure patterns, specifically leading to lower expenditure and higher GCs as consumers curtail their meat expenditure and switch to alternative products. Figure 3h/i and Fig. 4h/i supports the notion of increased prevalence of meat alternatives and fruit/vegetables in people’s diets, with an increasing proportion of expenditure and decreasing GC across all geodemographic groups. For example, in 2008 group 5r (London Cosmopolitan) spent 22.8% of their food budget on fruit and vegetables, increasing to 24.7% in 2017. Despite this increased proportion of expenditure on fruit, vegetables and meat alternatives, a universal decrease in the proportion of total expenditure on meat is not evident. While some meat categories do exhibit a trend towards reduced expenditure share and increased inequality (Fig. 3/Fig. 4b, d & e), this appears to be due to product specific drivers (as discussed for lamb) and is not ubiquitous across all meat categories. Some processed and red meat product categories which may be expected to see changes in expenditure/GC due to changing attitudes (e.g. sausages, other preserved and processed meat) actually display a rising or stable expenditure share and GCs. This suggests that, up to 2017 (the last year there is data available for this study), recent increases in environmental awareness and media coverage of plant-based diets have not had a widespread impact on overall meat expenditure patterns. Indeed it is still a small minority of the population that have diets completely free of meat, with a 2016 poll carried out on behalf of the Vegan Society finding 3.25% of adults never eat meat (Ipsos MORI 2016) whilst a 2017 survey by the Food Standards Agency found 3% of the 3118 respondents identified as vegetarian and 1% stated they were vegan (Bates et al. 2017). These findings are also in line with a recent review of consumer attitudes and behaviours, finding consumers who have changed their meat intake for ecological concerns are a small minority (Sanchez-Sabate and Sabaté 2019). This is a fast-changing area of research with an ever-increasing amount of information and guidance available (e.g. Willett et al. 2019). The framework described in this study can be easily applied to new releases of the Living Cost and Food survey as they become available, allowing the most recent trends to be assessed.

## Targeted Policy Interventions

The meat industry is a contentious component of the food system, with ongoing debate surrounding environmental impacts, human health and its importance to the economy. Any policy interventions designed to tackle these issues need to be designed to have maximum effect on those who will benefit from the policy whilst minimising negative impacts on others. Such an approach has recently been applied to the Scottish alcohol sector where minimum unit pricing was introduced in 2018. The policy was designed to specifically target harmful drinkers whilst minimising the negative impacts of price increase on those who drink within the lower risk guidelines. Early indications suggest that the policy is working, with reductions of purchased alcohol occurring in the households that bought the most alcohol (O’Donnell et al. 2019). It is estimated that once the policy reaches full effect, annual hospital admissions will fall by 2000 and alcohol related deaths will fall by 120 (Angus et al. 2016).

Although this study does not aim to recommend any specific policies, the methodology presented herein may be used by policy makers to ensure the correct consumer groups are targeted by any future interventions. This can be demonstrated by considering health concerns related to red and processed meat over-consumption, such as bowel cancer. Individuals who already consume relatively small quantities of red meat are generally at lower risk, with the World Cancer Research Fund (WCRF) recommending consumption of up to 500 g per week (WCRF 2018b). These individuals would therefore have little health benefit from such a policy intervention. Furthermore, red meat is often an important dietary source of protein and essential nutrients (Wyness 2016) so in some cases, a reduction in meat consumption may compromise the nutritional adequacy of a diet if appropriate substitutions are not made (WCRF 2018a). Thresholds may differ depending on the specific aim of the policy, with consumption of up to 98 g of red meat per week suggested for a sustainable diet (EAT-Lancet 2019). This study provides a framework for targeting the correct geodemographic groups in relation to any given threshold.

Indirect negative impacts to the industry also need to be considered as reduced consumption and demand may lead to unemployment in the sector which has been proven to be detrimental to health (Dorling 2009), and even increase the risk of mortality (Roelfs et al. 2011). A comparable situation occurred during the decline of coal mining in the UK, where employment in the sector fell from 240,000 in 1981 to just 6000 by 2011 (Aragón et al. 2018). Areas which experienced widespread mine closure were still suffering from unemployment and deprivation over 20 years later (Beatty et al. 2007), highlighting the potential risks of widespread change in a particular industry and the need for relevant policy to minimise negative impacts.

Figure 5 demonstrates differences in expenditure patterns between geodemographic groups, which can be used to identify those who may benefit most and least from a particular policy intervention. For example, members of super-group 4 (ethnically diverse and metropolitan living) spend comparatively little on red and processed meat products, with high expenditure inequality (high GC). As such, it may not be beneficial for policy interventions aiming to reduce health risks to target those in super-group 4 (Ethnically diverse and metropolitan living). Furthermore, the geodemographic segmentation of consumer attitudes data (Fig. 6) offers an insight into how consumers may respond to policies based on their attitudes. For example, group 5r (London cosmopolitan) have the most environmentally friendly attitudes of any group (Fig. 6), so a policy leading with environmental sustainability might be one way to make any interventions more likely to succeed for this group. Conversely geodemographic group 3r (Countryside Living) have less environmentally friendly attitudes, so may be less likely to change their behaviour based on solely environmentally focussed policies.

Whilst this study uses proportion of total household food expenditure and GC to explore differences between geodemographic groups, actual consumption values could be used if suitable data were available. This would allow average consumption values to be compared with threshold levels to quantify risk of each geodemographic group. For example, the WCRF suggests consumption of up to 500 g red meat per week (WCRF 2018b) from a purely health perspective whilst a threshold of 98 g per week is suggested for a sustainable diet (EAT-Lancet 2019). Although it is beyond the scope of this study to recommend specific thresholds, it provides a framework for identifying geodemographic groups who consume above and below a given value.

## Conclusions

The future of the meat industry will require the management of important trade-offs between economic, environmental and health concerns which we highlight in this paper. A resilient industry is important for jobs and for sustainable provision of food, however a move to reduced consumption would be positive for the health of certain geodemographic groups and for the environment. Although detailed modelling of the mechanisms for doing this are outside the scope of this paper, we show how geodemographic classification can be used to assess the current and recent resilience of the meat industry. By grouping expenditure data by geodemographic clusters, it is possible to detect trends, patterns and fluctuations which would be difficult to identify at the original Local Authority District level (*n* = 380).

Food expenditure patterns are shown to vary both temporally and by geodemographic group. Whilst the expenditure patterns of some food categories such as sausages have remained relatively consistent between 2008 and 2017, other such as lamb show a trend towards a reduced proportion of expenditure and increasing inequality. Short term fluctuations (of both expenditure proportion and GC) are also visible in the temporal record for certain categories (e.g. for chicken in 2012–14), potentially due to product specific price variability, price elasticities or disease scare (Pritchett et al. 2007). In addition to temporal trends and fluctuations, the geodemographic groups 4r (Ethnically diverse and metropolitan living) and 5r (London cosmopolitan) spend a relatively low proportion of their food budget on a number of red and processed meat categories (e.g. beef, pork, bacon, sausages, and other processed/preserved meats), whilst also exhibiting high levels of expenditure inequality for these food categories. Although the expenditure pattern of these two groups is similar, different drivers are likely to be responsible. Attitudes towards the environment and climate change is likely influencing the expenditure patterns of group 5r whilst the prominence of communities who do not eat meat for religious or cultural reasons and financial constraints is probably driving the patterns observed in group 4r.

The proportion of expenditure on meat alternatives and fruit and vegetables has increased over the study period, possibly due to the growing awareness and publicity of environmental and health issues. Although the proportion of expenditure on some meat categories has also reduced (e.g. pork, lamb, bacon), the expenditure patterns of other meat categories remained consistent or increased (e.g. sausages, other processed meats), suggesting environmental and health issues are not currently causing a universal reduction in meat expenditure.

The role of socio-demographics in the context of economic, environmental and health issues is of critical importance (Dorling and Gietel-Basten 2017; James 2019), with it being increasingly common for ‘big data’ approaches to provide the basis for decision making (Birkin et al. 2020). Whilst this study is focussed on expenditure patterns within the meat industry, the framework could equally be used for variables associated with deprivation, health and income distribution. This could be a valuable tool for policy makers in the arena of sustainable development where a major objective is to reduce social and environmental injustice (HM Government 2005). To achieve this, robust information on current inequalities (and trends) is vital for devising sustainable development policies. Measuring inequalities between geodemographic regions is of relevance as development strategies are often best pursued at local area or community level.

## Appendix 1

**Table 2:** Food categories and products used in this study. See James et al., (2019) for a full description of the coding framework

Description	COICOP code	Example products included
Pork (fresh, chilled or frozen)	1.1.2.2.1	Polony, pork belly, pork ribs, pork joint, pork steaks, pork leg, pork, tenderloin, pork, pork filet, pork mince, pork spare ribs, pork, shoulder, pork chops, spare ribs
Beef (fresh, chilled or frozen)	1.1.2.1.1	Beef mince, beef, braising steak, brisket, chuck steak, chump steak, grill steaks, ground beef, joint beef, mince, minced beef, minced, meat, rib steaks, rump steak, shin of beef, silverside beef, sirloin, steak, steak, stewing steak, topside, veal
Lamb (fresh, chilled or frozen)	1.1.2.3.1	Gigot, kebabs (unspecified), lamb chops, lamb cutlets, lamb kebabs, lamb leg, lamb breast, lamb steaks, lamb, lamb fillet, leg of lamb, loin chops, minced lamb, shoulder of lamb, stewing lamb
Poultry (fresh, chilled or frozen)	1.1.2.4.1	Chicken legs, chicken pieces, chicken (frozen), chicken, chicken, joints, chicken breasts, chicken stirfry (uncooked), chicken wings, duck, grouse, goose, guinea fowl, ostrich, partridge, pheasant, pigeon, quail, turkey steaks, turkey, turkey mince, turkey escalopes
Bacon and Ham (uncooked)	1.1.2.5.2	Bacon rashers, bacon, bacon chops, bacon joint, forehock joint, gammon steaks, gammon, ham
Sausages	1.1.2.5.1	Beef sausages, cumberland sausage, deli type sausages, garlic, sausage, kabanos (peppery sausage), polony (spicy sausages), pork, sausage, pork chipolatas, sausage meat, sausages (frozen), sausages, Wallbangers (sausages), bierwurst, chorizos, frankfurters (vacuum packed), german sausage, hotdog sausages, liver sausage, salami, saveloys
Other preserved or processed meat and meat preparations	1.1.2.6.1	Bacon & ham (canned), bacon burgers, baked beans & burgers, (tinned), beans & bacon burgers, beef (canned), beefburgers, burgers, cheeseburgers, chicken (tinned), chicken burgers, corned beef, cottage pie, dale steaks, frankfurters (tinned), frozen burgers,,ham & pork (tinned), ham (tinned), hamburgers, hot dogs (tinned), lamb burgers, luncheon meat, meat (tinned), meat balls, meat pate (tinned), meat pie fillings (tinned), pork burgers, poultry (tinned), sausages (tinned), sausage & beans (tin), sausage burgers, shepherd’s pie, sheppie, spam, steak burgers, steaklets, stewed steak (canned), tinned meat, toast toppers, turkey (tinned), turkey burgers, bacon turnover, beef & vegetable pie, beef & onion pie, beefsteak, pie, chicken & mushroom pie, chicken pie, chicken & vegetable pies, cornish pasties, cumberland pie, McColgans (Northern Ireland meat pies), meat pudding, meat pastie, meat & potato pie, meat pie, meat samosa, Melton Mowbray pie, mince pie (meat), minced beef & onion pie, pastie, pork pie, sausage pie, sausage rolls, sausage & onion pie, steak pudding, steak & mushroom pie, steak pie, steak pie (tinned), steak & kidney pie, veal & ham pie, bacon and ham (cooked), bacon joints (cooked), beef (cooked), brisket (cooked), chicken (cooked), chicken (smoked), cold meat (cooked), cooked meat, corned beef, crunchy chicken, Danish ham (cooked), gammon (cooked), garlic ham, ham (sliced), ham (baked), ham (cooked), ham (unspecified), ham (boiled), ham (smoked), ham (roast), honey roast ham (cooked), lunch tongue, luncheon meat, parma ham (cooked), pastrami, pepperoni, pork (cooked), pork slices, pork luncheon meat, pork roll, pork and ham roll, pressed tongue, salt beef (pre-packed), turkey (roast), turkey (cooked), turkey (sliced),turkey ham, turkey roll, turkey and bacon loaf, veal (cooked), beef chow mein, beef curry, beef goulash (frozen), beef hotpot, beef risotto, beef stroganoff (frozen), black pudding, burger in a bap, cannelloni, chicken tikka (cooked), chicken/broccoli ready meal, chicken casserole, chicken chasseur, chicken chow mein, chicken en croute, chicken & veg roll (frozen), chicken masala (cooked), chicken curry (cooked), chicken dippers, chicken in white sauce, chicken pasta (ready to eat), chicken stir fry (ready meal), chicken supreme, chicken curry (ready to eat), chicken in sauce (cooked), chicken & mushroom golden moments, chicken noodles, chilli con carne, corn beef hash, crispy pancakes with meat, faggots, haggis, ham & cheese savoury bakes, haslet, hot pot (ready meal), irish stew, kebabs, lasagne (ready to eat), lasagne (frozen), meat balls, meat based ready meals, meat curry, meat loaf, meat salads (prepacked), meat stirfry, mince ready meal, minced beef frizbees (frozen), minced beef casserole, moussaka, pepperami roll, pork scratchings, potato chicken & broccoli meal, ready made chicken & orange, roast beef in gravy, sausages & mash, savoury duck, scotch eggs, Southern fried chicken, spaghetti bolognese, sweet & sour pork, tagliatelle carbonara, toad in the hole, vienna steak, white puddings in batter, potato chicken & broccoli meal, ready made chicken & orange, roast beef in gravy, sausages & mash, savoury duck, scotch eggs, Southern fried chicken, spaghetti bolognese, sweet & sour pork, tagliatelle carbonara, toad in the hole, Vienna steak, white puddings in batter, cajun chicken, chicksticks, chicken drumsticks, chicken nuggets, chicken kiev, chicken nibbles, chicken fingers, chicken in sauce, golden drummers, pasta and ham salad
Other preserved or processed vegetables	1.1.7.6.1	Artichoke (hearts, tinned, jarred, other processed), asparagus (tinned), avocado dip (frozen), bamboo shoots (tinned), beansprouts (tinned), beetroot (cooked), beetroot (pickled), broccoli & cheese bake, carrots (tinned), celery (tinned), chestnuts (canned), chopped tomatoes, corn niblets, creamed mushrooms, creamed tomatoes, dahl (ready to eat), florida salad, garlic pate, garlic puree, gherkins, green beans (tinned), guacamole, lava bread, mixed vegetables (tinned), mushrooms (tinned), mushroom pate, mushroom ravioli, passata (tomato), peas (tinned), pease meal, pickled cucumbers, pickled onions, pickles, processed peas (tinned), pulses (tinned), red cabbage (jar), sauerkraut, sundried tomatoes in oil, sweetcorn (tinned), tomatoes (bottled), tomatoes (tinned), tomato puree, vegetable curry & rice, vegetable lasagne, vegetable salad (tinned), vegetables (tinned), vegetables (bottled), vegetable based frozen ready meals, vegetable spring rolls, vegetable stirfry, vegetable products (frozen), vegetable purees, vegetable slices, water chestnuts (tinned), baked beans (tinned), beanfeast, beans (tinned), beans cuisine (tinned), boiled beans (tinned), broad beans (tinned), butter beans (tinned), chick peas (tinned), chilli beans (tinned), falafel, houmous, hummus, kidney beans (tinned), lentils (tinned), lima beans (tinned), marrowfat peas (tinned), mushy peas (tinned), pease pudding, refried beans, cauliflower cheese, cheese & vegetable roll, cheese salad, coleslaw, egg salad, nut salad, potatoes & bean fritter, ratatouille (ready to eat), three bean salad, vegetable crowns, vegetable curry (tinned), vegetable lasagne (frozen), vegetable pate, vegetable pie, vegetable hot-pot, cheese & onion bakes, cheese & onion fingers, cheese pasties, leek & mushroom pie, vegetable samosa, vegetarian pasties, bean curd, miso, nut cutlets, Nuttolene, powdered vegetable burger mix, Quorn, Quorn crispbakes, soya mince, soya protein, tofu, veda, vegebangers, vegeburgers, vegerissoles, vegetable mince, vegetable sausages, vegetarian mince, vegetarian sausages, vegetarian mixes, onion bhaji, pakoras, spring rolls, rice and vegetables, pickled eggs
All fruit and vegetables	1.1.6.1.1	Citrus fruits – fresh: citrus fruits, clementines, grapefruit, kumquat, lemon, lime, mandarins, minneola, oranges, ortaniques, satsumas, tangerines, ugli fruit
	1.1.6.2.1	Bananas – fresh
	1.1.6.3.1	Apples – fresh: apples, cooking apples, toffee apples
	1.1.6.4.1	Pears – fresh
	1.1.6.5.1	Stone fruits – fresh: apricots, avocados, cherries, damsons, dates, greengages, lychees, mango, nectarines, peaches, plums, stuffed olives
	1.1.6.6.1	Berries – fresh: blackberries, blackcurrants, blueberries, cranberries, gooseberries, grapes, loganberries, mulberries, raspberries, red currants, strawberries, tayberries
	1.1.6.7.1	Other fresh, chilled or frozen fruits: figs, fresh fruit salad, fruit, fruit (frozen), fruit products (frozen), guava, kiwi fruit, melon, melon balls (frozen), papaya, passion fruit, paw-paw, peach halves (frozen), persimmon, pineapple, pomegranates, prickly pear, quinces, raspberries (frozen), rhubarb, sharon fruit, star fruit, strawberries (frozen), watermelons
	1.1.6.8.1	Dried fruit and nuts: almonds (flaked/ground/whole), almond paste, apricots (dried), bananas (dried), brazil nuts, capers, cashew nuts, cherries (glace), chestnuts, chestnut puree, chopped nuts, cob nuts, coconut (fresh), currants (dried), crystallised fruit, dates (dried), dried figs, dried fruit and nuts, dried mixed fruit, fruit (dried), edible seeds, hazelnuts, nuts & raisins, nuts, peanuts salted/dry/roasted, peaches (dried), pineapple rings (dried), pine kernels, pistachios, prunes (dried), pumpkin seeds, raisins (dried), salted nuts, Savour Mince, sultanas (dried), tahini (nuts), vegetarian nut products, walnuts, water chestnuts, mincemeat, dessicated coconut, ground almonds, marzipan, sesame seeds, sunflower seeds.
	1.1.6.9.1	Preserved fruit and fruit-based products: tinned), fruit cocktail (tinned), fruit (bottled), fruit compote, fruit (tinned), fruit pie fillings, fruit salad (tinned), glace cherries, grapefruit (tinned), mandarins (tinned), olives (bottled), peaches (tinned), pears (tinned), pie fillings, pineapple (tinned), prunes (tinned), rhubarb (tinned), satsumas (tinned), stem ginger (tinned), strawberries (tinned)
	1.1.7.1.1	Leaf and stem vegetables (fresh or chilled): artichokes, globe artichokes, asparagus, bamboo shoots, beansprouts, celeriac, celery, chicory, corn on the cob, cos lettuce, cress, crispy seaweed, endive lettuce, iceberg lettuce, leafy salads, lettuce, little gem lettuce, mustard and cress, radicchio lettuce, salad-mixed, spinach, spinach beet, sprouting broccoli, sweetcorn, turnip tops, vine leaves, watercress, web lettuce, basil (fresh), clove of garlic (fresh), coriander (fresh), herbs (fresh/potted), garlic (fresh), ginger (fresh), lemongrass (fresh), mint (fresh), parsley (fresh), pimentoes, rosemary (fresh), sage (fresh), thyme (fresh)
	1.1.7.2.1	Cabbages (fresh or chilled): broccoli, brussel sprouts, cabbages, calabreses, cauliflower, Chinese leaves, greens, headed broccoli, kale, pak choi (chinese cabbage), red cabbage, savoy cabbage, sea kale, spring cabbage, spring greens, sprouts, white cabbage
	1.1.7.3.1	Vegetables grown for their fruits (fresh, chilled or frozen): aubergine, beans, broad beans, capsicums, chillis, courgettes, cucumber, french beans, green bananas, green beans, green peppers, ladies fingers, mange tout (peas), marrow, mixed peppers, okra, peas, peppers, plantains, pumpkin, red peppers, runner beans, sugar snap (peas), tomatoes, beans (frozen), broad beans (frozen), cut beans (frozen), mange tout (frozen), mushy peas (frozen), peas (frozen), petit-pois (frozen), sliced beans (frozen)
	1.1.7.4.1	Root crops, non-starchy bulbs and mushrooms (fresh, chilled or frozen): Jerusalem artichoke, beetroot (raw), carrots, cassava, crudites, fennel, fresh vegetables (not specified), horseradish, leeks, mixed vegetables, mushrooms, onions, oyster mushrooms, parsnips, radishes, root ginger, scallions, shallots, spring onions, stewpack (mixed vegetables), stir-fry mix, sweet potatoes, swedes, syboes, turnips, vegetables, yams, asparagus (frozen), beansprouts (frozen), broccoli (frozen), brussel sprouts (frozen), cabbage (frozen), carrots (frozen), cauliflower (frozen), corn on the cob (frozen), frozen herbs, mix fantastic (frozen) in NI, mixed vegetables (frozen), mixed peppers (frozen), mushrooms (frozen), onion rings (frozen), parsley (frozen), ratatouille (frozen), spinach (frozen), sprouts (frozen), stir-fry vegetables (frozen), swede (frozen), sweetcorn (frozen), vegetables (frozen)
	1.1.7.5.1	Dried vegetables: Aduki beans (dried), air-dried peppers, bean mix (dried), black-eyed beans (dried), broth mix, butter beans (dried), chick pea flour, dahl (dried), haricot beans (dried), kidney beans (dried), lentils (dried), marrowfat peas (dried), masoor, mixed barley, peas (dried), pulses, soya beans, split peas (dried), sundried tomatoes (not in oil), vegetables (dried)
	1.1.7.6.1	Other preserved or processed vegetables: tinned), avocado dip (frozen), bamboo shoots (tinned), beansprouts (tinned), beetroot (cooked), beetroot (pickled), broccoli & cheese bake, carrots (tinned), celery (tinned), chestnuts (canned), chopped tomatoes, corn niblets, creamed mushrooms, creamed tomatoes, dahl (ready to eat), florida salad, garlic pate, garlic puree, gherkins, green beans (tinned), guacamole, lava bread, mixed vegetables (tinned), mushrooms (tinned), mushroom pate, mushroom ravioli, passata (tomato), peas (tinned), pease meal, pickled cucumbers, pickled onions, pickles, processed peas (tinned), pulses (tinned), red cabbage (jar), sauerkraut, sundried tomatoes in oil, sweetcorn (tinned), tomatoes (bottled), tomatoes (tinned), tomato puree, vegetable curry & rice, vegetable lasagne, vegetable salad (tinned), vegetables (tinned), vegetables (bottled), vegetable based frozen ready meals, vegetable spring rolls, vegetable stirfry, vegetable products (frozen), vegetable purees, vegetable slices, water chestnuts (tinned), baked beans (tinned), beanfeast, beans (tinned), beans cuisine (tinned), boiled beans (tinned), broad beans (tinned), butter beans (tinned), chick peas (tinned), chilli beans (tinned), falafel, houmous, hummus, kidney beans (tinned), lentils (tinned), lima beans (tinned), marrowfat peas (tinned), mushy peas (tinned), pease pudding, refried beans, cauliflower cheese, cheese & vegetable roll, cheese salad, coleslaw, egg salad, nut salad, potatoes & bean fritter, ratatouille (ready to eat), three bean salad, vegetable crowns, vegetable curry (tinned), vegetable lasagne (frozen), vegetable pate, vegetable pie, vegetable hot-pot, cheese & onion bakes, cheese & onion fingers, cheese pasties, leek & mushroom pie, vegetable samosa, vegetarian pasties, bean curd, miso, nut cutlets, Nuttolene, powdered vegetable burger mix, Quorn, Quorn crispbakes, soya mince, soya protein, tofu, veda, vegebangers, vegeburgers, vegerissoles, vegetable mince, vegetable sausages, vegetarian mince, vegetarian sausages, vegetarian mixes, onion bhaji, pakoras, spring rolls, rice and vegetables, pickled eggs
	1.1.7.7.1	Potatoes: baking potatoes, new potatoes, potatoes (fresh)
	1.1.7.8.1	Other tubers and products of tuber vegetables: american fries, chips, croquette potatoes, fries (frozen), frozen chips, frozen roast potatoes, hash browns, instant potato, jacket potatoes (ready to heat), jacket scollops, oven chips, potato (dried), potato fritters, potato pies, potato rings, potato salad, potato croquettes, potato waffles, potato scones, potato skins, potato cakes, potatoes (bottled), potatoes (tinned), potatoes (sauted), Smash, southern fries, crisps (potato sticks), crisps, Hula Hoops, Kettle chips, mini chips, Pringles, potato crisps, potato snacks, potato squares, potato crunchies, Quavers, salt and shake, stackers

## Appendix 2

**Table 3:** The 59 statistics from the 2011 Census that were used to create the 2011 Area Classification for Local Authorities**.** Adapted from https://www.ons.gov.uk/methodology/geography/geographicalproducts/areaclassifications/2011areaclassifications/abouttheareaclassifications

Demographic structure	House-hold composition	Housing	Socio-Economic	Employment
•% of persons living in a communal establishment •Number of persons per hectare •% of persons aged 0–4 •% of persons aged 5–14 •% of persons aged 25–44 •% of persons aged 45–64 •% of persons aged 65–89 •% of persons aged 90+ •% of persons aged 16+ who are single •% of persons aged 16+ who are married or in a registered same-sex civil partnership •% of persons aged 16+ who are divorced or separated •% of persons who are white •% of persons who have mixed ethnicity or are from multiple ethnic groups •% of persons who are Asian/Asian British: Indian/Pakistani/Bangladeshi •% of persons who are Asian/Asian British: Chinese and Other •% of persons who are Black/African/Caribbean/Black British •% of persons who are Arab / are from another ethnic group •% of persons whose country of birth is the UK / Ireland •% of persons whose country of birth is in the old EU (pre 2004 accession countries) •% of persons whose country of birth is in the new EU (post 2004 accession countries) % of persons whose main language is not English and cannot speak English well or at all	• % of households with no children • % of households with non-dependent children • % of households with full-time students	• % of households who live in a detached house or bungalow • % of households who live in a semi-detached house or bungalow • % of households who live in a terrace or end-terrace house • % of households who live in a flat • % of households who live in a caravan or other mobile or temporary structure • % of households who own or have shared ownership of property • % of households who are social renting • % of households who are private renting • % of households who have one fewer room or less rooms than required	• Individuals day-to-day activities limited a lot or a little (Standardised Illness Ratio) • % of persons providing unpaid care • % of persons aged 16+ whose highest level of qualification is Level 1, 2 or Apprenticeship • % of persons aged 16+ whose highest level of qualification is Level 3% of persons aged 16+ whose highest level of qualification is Level 4 and above • % of persons aged 16+ who are schoolchildren or full-time students • % of households with 2 or more cars or vans • % of persons aged 16–74 who use public transport to get to work • % of persons aged 16–74 who use private transport to get to work • % of persons aged 16–74 who walk, cycle or use an alternative method to get to work	• % of persons aged 16–74 who are unemployed • % of employed persons aged 16–74 who work part time • % of employed persons aged 16–74 who work full-time • % of employed persons aged 16–74 who work in agriculture, forestry or fishing • % of employed persons aged 16–74 who work in mining, quarrying or construction • % of employed persons aged 16–74 who work in manufacturing • % of employed persons aged 16–74 who work in energy, water or air conditioning supply • % of employed persons aged 16–74 who work in the wholesale and retail trade • % of employed persons aged 16–74 who work in the transport or storage industries • % of employed persons aged 16–74 who work in accommodation or food service activities • % of employed persons aged 16–74 who work in information and communication or professional, scientific and technical activities • % of employed persons aged 16–74 who work in financial, insurance or real estate • % of employed persons aged 16–74 who work in administrative or support service • % of employed persons aged 16–74 who work in public administration or defence • % of employed persons aged 16–74 who work in education • % of employed persons aged 16–74 who work in human health and social work

## Appendix 3

Pen portraits for the 2011 Area Classification for Local Authorities (Super-groups only). Adapted from https://www.ons.gov.uk/methodology/geography/geographicalproducts/areaclassifications/2011areaclassifications/penportraitsandradialplots

**1r – Affluent England**

51 local authorities – 10.3% of UK population, population density 3.7, median age 41 years. The population of this supergroup typically live largely in counties in England near to and around London – Buckinghamshire, Hampshire, Hertfordshire, Kent, Oxfordshire and Surrey. Residents are much more likely to live in detached housing and to own their own property. The supergroup has an above average ethnic mix and below average number of UK and Irish born residents. Residents are far more likely to be represented in the 5 to 14 years age group than nationally. Rates of divorce or separation are lower than nationally and the proportion of persons aged 16 years and over with higher qualifications is above the national average. Unemployment rates are noticeably below the national average and for employed residents, they are more likely to work in the information and communication industries than nationally, more likely to work full-time, and are more likely to travel to work using public transport, though households owning two or more cars are also more prevalent than nationally.

**2**^**r**^ **– Business, education and heritage centres**

35 local authorities – 14.4% of UK population, population density 17.4, median age 35 years. The population of this supergroup live within larger cities throughout the UK, with either country and/or regional importance. Residents are more likely to live in either flats or terraces and to privately rent their home. The supergroup has an above average ethnic mix, with an above average proportion of residents born in other EU countries. A result of this is that households are less likely to speak English or Welsh as their main language. Those in employment are more likely to be working in the accommodation or food service activities and education sector and to use public transport to travel to work. Compared with the UK, educational qualifications are higher, though unemployment is marginally higher.

**3r – Countryside living**

83 local authorities – 15.2% of UK population, population density 0.7, median age 46 years. The population for this supergroup are characterised by living in rural areas with a low population density and a much higher median age (46 years) compared with the UK as a whole (39 years). Residents are more likely to own their own home and to live in a detached property. Unemployment and qualification levels are both below the national average. Households are more likely to own two or more vehicles and to use private transport to travel to work. Part-time working is also more prevalent than generally in the UK. Workers are far more likely to be employed in the agriculture, forestry and fishing industry, and the accommodation or food service activities industries than workers in the UK generally.

**4r – Ethnically diverse metropolitan living**

19 local authorities – 9.4% of UK population, population density 46.5, median age 33 years. The population of this supergroup tend to be live in Inner and Outer London Boroughs. Areas outside London covered by this supergroup include Birmingham, Leicester, Luton and Slough. The major characteristic of these areas is that they have a high proportion of residents from non-White ethnic groups. Population density is high, as is overcrowding (households who have on average fewer or less rooms than required) and there is a relatively young population age structure. Households are more likely to reside in terrace housing or flats, either socially-rented or privately-rented, than households generally. Unemployment is higher than recorded nationally. Compared with the UK as a whole, workers in the administrative or support services industry are most over-represented and workers are more likely to use public transport to get to work.

**5r – London cosmopolitan**

12 local authorities – 4.2% of UK population, population density 106.8, median age 32 years. The population of this group is located within 12 Inner London boroughs. The areas of London covered by this supergroup are characterised by a very high population density and a relatively low median age (32 years). All non-White ethnic groups have a higher representation than the UK as a whole, especially people of Black or Arab ethnicity, with an above average proportion of residents born in other EU countries. Residents are more likely to live in flats and are more likely to rent. A higher proportion of people use public transport to get to work, with lower car ownership and higher unemployment. Those in employment are more likely to work in the information and communication, and the financial, insurance or real estate industries.

**6r – Services and industrial legacy**

57 local authorities – 15.3% of UK population, population density 4.7, median age 41 years. The population of this group is predominately in the Central Belt in Scotland, northern England and south Wales – all traditional mining areas. There is a much lower representation of ethnic minority groups than for the UK as a whole and a higher than average proportion of residents born in the UK and Ireland. Rates of divorce and separation are above the national rate. Households are more likely to have children and are more likely to live in semi-detached or terraced properties and to socially rent. There is a smaller proportion of people with higher-level qualifications, with rates of unemployment above the national level. Those in employment are more likely to be employed in the energy, water or air conditioning industries, manufacturing industries, and the mining, quarrying or construction industries. Workers are more likely to use private transport to travel to work than nationally.

**7r – Town and country living**

79 local authorities – 16.1% of UK population, population density 2.0, median age 42 years. This supergroup is represented within all countries of the UK and English regions with the exception of the North East and London regions. The population density (at 2.0 persons per hectare) is below the UK as a whole (at 2.6 persons per hectare). There is a low proportion of persons by minority ethnic group and a relatively high proportion of residents were born in the UK or Ireland. Compared with the UK as a whole, the population is more likely to be aged 45 years and over, and with a higher median age (42 years). Residents are more likely to live in detached or semi-detached properties, to own their home and to use private transport for travel to work, with higher car ownership. Residents also have higher levels of educational attainment. People are more likely to work in manufacturing industries.

**8r – Urban settlements**

55 local authorities – 15.0% of UK population, population density 12.6, median age 38 years. The population of this supergroup are confined to the nine English regions and Wales (Newport) only. The areas are characterised by a slightly younger age structure than nationally, with higher proportions of all groups aged 45 and under (covering the age groups 0 to 4 years, 5 to 14 years and 25 to 44 years). Ethnic groups are over-represented compared with the national picture and households are more likely to live in semi-detached or terraced housing. Adults generally have lower qualifications than nationally and are more likely to be unemployed. Residents who are employed are more likely to work in the wholesale and retail trade, transport and storage, and administrative and support services industries. Workers are more likely to commute using public transport and car ownership is lower than nationally.
